# Long-Term Outcomes of Dental Rehabilitation and Quality of Life after Microvascular Alveolar Ridge Reconstruction in Patients with Head and Neck Cancer

**DOI:** 10.3390/jcm13113110

**Published:** 2024-05-25

**Authors:** Katharina Zeman-Kuhnert, Alexander J. Gaggl, Gian B. Bottini, Joern Wittig, Georg Zimmermann, Christoph Steiner, Wanda Lauth, Christian Brandtner

**Affiliations:** 1 Department of Oral and Maxillofacial Surgery, University Hospital of Salzburg, Paracelsus Medical University, 5020 Salzburg, Austria; a.gaggl@salk.at (A.J.G.); g.bottini@salk.at (G.B.B.); j.wittig@salk.at (J.W.); c.steiner@salk.at (C.S.); c.brandtner@salk.at (C.B.); 2 Team Biostatistics and Big Medical Data, IDA Lab Salzburg, Paracelsus Medical University, 5020 Salzburg, Austria; georg.zimmermann@pmu.ac.at (G.Z.); wanda.lauth@pmu.ac.at (W.L.)

**Keywords:** head and neck cancer, oral cancer, dental rehabilitation, alveolar ridge reconstruction, quality of life, oral health-related quality of life, reconstructive surgery, OHIP-49, Short Form-36

## Abstract

**Background/Objectives**: Dental rehabilitation after extended tumour resection and jaw reconstruction is challenging. The present study aimed to report the prosthetic outcome and quality of life (QoL) in patients with head and neck cancer (HNC) after microvascular alveolar ridge reconstruction. **Methods**: The prosthetic outcomes of all consecutive patients with HNC who underwent microvascular alveolar ridge reconstruction at the University Hospital Salzburg between 2011 and 2018 were investigated. Oral health-related QoL (OHrQoL) and overall QoL were assessed using the validated Oral Health Impact Profile-49 (OHIP-49) and Short Form-36 questionnaires. **Results**: During the study period, 115 consecutive patients with head and neck cancer underwent microvascular jaw reconstruction. Among them, 23.3% and 27.4% received conventional tissue-borne prostheses and implant-supported prostheses, respectively, while 48.7% did not undergo dental rehabilitation. The prosthetic outcome was not associated with tumour stage (*p* = 0.32). Oral health-related quality of life (OHrQoL) was best in patients with implant-supported dental rehabilitation (OHIP-49 median score = 7) and worst in those with conventional removable dentures (OHIP-49 median score = 54). The corresponding OHIP-49 median score for patients who could not undergo dental rehabilitation was 30.5. All Short Form-36 subscale scores were equal to or higher than the malignancy norm scores. **Conclusions**: After microvascular jaw reconstruction, approximately one-third of the HNC patients received adequate implant-supported dental rehabilitation. However, the risk of dental rehabilitation failure was 50%. The different prosthetic outcomes affected OHrQoL, but not overall QoL.

## 1. Introduction

Head and neck cancer (HNC) is the sixth most common cancer globally, accounting for more than 945,000 new cases and 480,000 deaths annually [1]. The overall incidence of HNC continues to rise, with a predicted 30% increase per year by 2030 [2].

Bone continuity defects in the mandible or maxilla following head and neck cancer resection result in the loss of soft tissues and bone. Inadequate reconstruction results in several complications, including facial asymmetry and disharmony, large oro-nasal and oro-antral communication, or impairments in speech, mastication, swallowing, and saliva retention [3,4,5]. Current microvascular reconstruction techniques can restore jaw continuity and improve orofacial aesthetics. However, mastication and speech may still be impaired because of a lack of functional dentition [5,6]. Therefore, dental restoration should be part of the overall functional rehabilitation of patients.

Nevertheless, achieving functional dental restoration after extensive HNC resection and oral cavity reconstruction is challenging. Unfavourable conditions such as insufficient bone height or poor soft tissue overlying the bone flap or graft make tissue-borne removable prostheses less viable. Patients with T3 and T4 tumours receive adjuvant radiotherapy, which may lead to xerostomia and tissue fibrosis. The irradiated mucosa often does not tolerate the friction and mechanical stress associated with conventional tissue-supported prostheses. In addition, xerostomia reduces the vacuum effect between the prosthesis and immobile underlying soft tissues [5,7].

To address these challenges, the placement of dental implants in reconstructed areas may offer a solution. Existing literature suggests that implant-supported prostheses offer HNC patients more effective dental rehabilitation than conventional removable prostheses [5,8,9,10]. However, implant-supported prostheses are not feasible in all cases and may be contraindicated, especially in patients receiving adjuvant therapies. Therefore, if the tissue-borne prosthesis fails, the patient will be left without suitable dental rehabilitation. In this context, the primary objective of this study was to evaluate the outcomes of dental rehabilitation in HNC patients who underwent microvascular jaw reconstruction over 10 years. Clinical records were analysed to determine whether tumour staging, management approaches, and complications prevented successful dental rehabilitation.

Reconstructive surgery aims to restore the form and function of the masticatory system and, crucially, improve the patient’s quality of life (QoL). Therefore, the presentation of the outcomes of dental rehabilitation in isolation, as performed in some previous studies, is insufficient. Thus, in the second part of this study, the oral health-related and overall QoL of the same group of patients was assessed. The survey aimed to incorporate the patients’ subjective perceptions alongside the objective analysis of alveolar ridge reconstruction and dental rehabilitation outcomes following HNC.

## 2. Materials and Methods

### 2.1. Outcome of Dental Rehabilitation after Alveolar Ridge Reconstruction

All patients with HNC who underwent microvascular segmental alveolar ridge reconstruction with a bone flap at the Department of Oral and Maxillofacial Surgery of the University Hospital Salzburg, Austria, between January 2011 and December 2018, were followed up from the day of reconstruction until the end of 2020, allowing a minimum follow-up time of 2 years. 

The course of dental rehabilitation and occurrence of complications requiring surgical treatment during the 10-year period were recorded. For dental implants placed in the microvascular flap, the implant loss and survival rates were analysed. 

In addition, information regarding age and sex, affected jaw, type of microvascular flap, type of reconstruction, and TNM classification was obtained from the patients’ medical records. Furthermore, perioperative tooth extractions were recorded.

### 2.2. Quality of Life Survey

From the defined cohort mentioned above, patients aged >18 years were invited to participate in a QoL survey conducted from June 2020 to July 2021. Written informed consent was obtained from all the patients. QoL was evaluated using two validated questionnaires. Oral health-related quality of life (OHrQoL) was assessed using the Oral Health Impact Profile-49 (OHIP-49), which consists of 49 items [11,12]. The response categories for the five-point Likert scale were “very often” (score 4), “fairly often” (score 3), “occasionally” (score 2), “hardly ever” (score 1), and “never” (score 0). The overall OHIP-49 score ranged from 0 to 196 (0 = best OHrQoL). The frequencies of problematic items (scores of 3 and 4) were recorded.

The 36-item Short Form Health Survey (SF-36) was used to assess the overall QoL [13,14]. The SF-36 items were categorised into eight subscales: “physical functioning” (PF), “role limitation due to physical health” (RP), “role limitation due to emotional problems” (RE), “vitality” (VT), “mental health” (ME), “social functioning” (SF), “bodily pain” (BP), and “general health” (GH). Scores for each subscale ranged from 0 to 100 (100 = best QoL). 

### 2.3. Statistical Analysis

Initially, associations between variables were descriptively analysed using Spearman’s correlations or counts and percentages, as appropriate. In the second exploratory step, Fisher’s exact test and generalisations were conducted to examine the associations among categorical variables. These tests were chosen because of the very small cell counts for some factor-level combinations. The Wilcoxon–Mann–Whitney test and a Kruskal–Wallis-type test were used to compare transplant sizes between groups [15]. The nonparametric relative effect was employed as the underlying effect measure, indicating a tendency toward larger values of the outcome variable in a particular subgroup compared to the entire study cohort. For two samples, the relative effect is defined as $p=P(X_{1}<X_{2})+\frac{1}{2}P\left(X_{1}=X_{2}\right)$, where *X_i_* ∼ *F_i_* (*X_i_* has distribution *F_i_*), *i* = 1, 2 are two independent random variables. For *N* independent samples (e.g., groups), the relative effect is defined as $p_{i}=\frac{1}{N}\sum_{l=1}^{N}\left[P\left(X_{l}<X_{i}\right)+\frac{1}{2}P\left(X_{l}=X_{i}\right)\right]$, where *X_i_* ∼ *F_i_*, *i* = 1, …, *N* [16]. 

The effect size used for the Wilcoxon–Mann–Whitney test is the correlation coefficient $r=\frac{Z}{\sqrt{n}}$, where *Z* ist the standardized *Z*-score and n the total number of observations. The values of r range between 0 and 1 and can be interpreted as r>0.10 to 0.3 (small effect), r>0.30 to 0.5 (moderate effect), and r≥0.5 (large effect) (i.e., the interpretation of the calculated r-value is the equivalent of that for Pearson’s correlation coefficient). For the Kruskal–Wallis test, the effect size eta squared was calculated as follows: $\eta^{2}\left[H\right]=\frac{H-k+1}{n-k}$, where *H* is the value determined in the Kruskal–Wallis test; *k* is the number of groups; n is the total number of observations. $\eta^{2}\left[H\right]$ also has values between 0 and 1 and can be interpeted as follows: $\eta^{2}>0.01\;\mathrm{to}\;0.06$ (small effect), $\eta^{2}>0.06\;\mathrm{to}\;0.14$ (moderate effect), and $\eta^{2}\geq 0.14$ (large effect). To interpret the results of the Fisher test, the phi coefficient $\Phi =\frac{a\cdot d-b\cdot c}{\sqrt{\left(a+b\right)\cdot \left(c+d\right)\cdot \left(a+c\right)\cdot \left(b+d\right)}}$ was calculated as an effect size for comparisons of interest. The interpretation of these values is similar to that for *r*. 

The two-sided significance level was set to alpha = 0.05 for all statistical tests. Statistical analyses were performed using the statistical software package R, version 4.0.2 [17].

## 3. Results

### 3.1. Outcomes of Dental Rehabilitation after Alveolar Ridge Reconstruction 

A total of 115 patients underwent segmental alveolar ridge reconstruction for HNC at University Hospital Salzburg between January 2011 and December 2018. Two patients lost to follow-up were excluded, leaving 113 patients for analysis. Among them, 72 were male, and the mean age at the time of alveolar ridge reconstruction was 60.6 years (range, 25.1–97.7 years). The mandible was reconstructed in 86 patients, while the remaining 27 underwent maxillary reconstruction.

Forty-one patients received a single bone flap, of which 30 received an iliac crest flap, five a fibula flap, three a scapula flap, two a medial femoral condylar flap, and one a combination of iliac crest and medial femoral condylar flap. 45 patients received an osteocutaneous bone flap, of which two were iliac crest flaps, 34 fibula flaps, six scapula flaps, two medial femoral condylar flaps, and one radialis flap. 27 patients had a double flap reconstruction, combining a bone flap with a soft tissue flap, such as the radial forearm flap (n = 3), anterolateral thigh flap (n = 12), lateral arm flap (n = 2), superior inferior epigastric artery flap (n = 6), saphenous perforator flap (n = 1), and groin flap (n = 3). Only once was an anterolateral thigh flap combined with a fibula flap; all other soft tissue flaps were used as a double flap together with the iliac crest flap. 

For further patient-specific information, please refer to Table 1.

#### 3.1.1. Prosthetic Outcomes

After alveolar ridge reconstruction, 27 (23.9%) patients received a conventional tissue-born prosthesis, with 16 (14.2%) receiving complete dentures and 11 (9.7%) receiving partial dentures. Implant-supported prosthetic restoration was achieved in 31 (27.4%) patients, including six (5.3%) with non-removable prostheses. Dental rehabilitation of the reconstructed alveolar ridge was not achieved in 55 (48.7%) patients. No statistically significant difference was observed between the maxilla and the mandible (*p* = 0.44; ϕ = 0.30). Furthermore, no statistically significant association was found between prosthetic outcome and tumour stage (*p* = 0.32; ϕ = 0.33).

Table 2 illustrates how the individual microvascular flaps were prosthetically restored. 

Approximately 14.2% of all patients underwent full mouth and 59.3% underwent partial mouth extractions peri-operatively. Furthermore, 26.5% had no extraction due to preoperative edentulism or cancer location in an edentulous jaw section (Table 3). 

#### 3.1.2. Dental Implants

Implants were inserted using templates after precise implant position planning based on 3D image data. Thirty-four patients underwent at least one implant placement. A total of 129 implants were placed, with a median of four implants per patient (range, 1–8 implants). Of the 34 patients, 10 (29.4%) experienced at least one implant loss. Overall, 23 (17.8%) of the 129 implants were lost; the median was two implants per patient (range, 1–5 implants). Implant survival in the study population was 82.2%. Sex did not influence implant loss (*p* = 0.22; ϕ = 0.21). 

All implants in the four patients with T1 tumours were preserved. Two of the 11 patients with T2 tumours lost a total of four implants (one and three pieces, respectively), four of the eight patients with T3 tumours lost a total of nine implants (one, one, two, and five pieces, respectively), and two of the seven patients with T4 tumours lost six implants (two and four pieces, respectively). These differences were not statistically significant (*p* = 0.38; ϕ = 0.31). One implant was lost from the four patients for whom the original tumour stage was not known.

Only three of the 34 patients who received implants remained without implant-supported dentures at the end of the study period. With the implant-supported prosthetics, 22 patients could eat solid food, three patients were on a soft diet, and six patients could only eat pureed food. 

#### 3.1.3. Reoperations and Complications following Alveolar Ridge Reconstruction

Planned soft tissue corrections for aesthetic reasons or prosthetic preparation were performed in 36 patients (31.9%). The overall reoperation/complication rate was 41.6%. Complications related to the osteosynthesis material (infected, exposed) occurred in 17 patients (15%), whereas complications related to the microvascular bone graft occurred in nine patients (8.0%). Eight patients (7.1%) lost their bone flaps; all eight patients received a new microvascular bone flap (7.1%). Complications associated with the soft tissue paddle (thrombosis and haemorrhage) occurred in six of the 45 patients (13.3%), but there was no paddle loss. Reoperations due to tumour recurrence at the site of origin were performed in 10 patients (8.8%), and reoperations related to recurrence/second tumour outside the area of bone reconstruction were performed in six patients (5.3%). Seven patients (6.2%) had other complications at the reconstruction site (e.g., fistula, pseudarthrosis, and wound dehiscence). Finally, three patients (2.7%) underwent reoperation for other complications near the reconstruction site (osteonecrosis and osteomyelitis). Tumour stage and the presence of lymph node metastases did not correlate with more frequent occurrence of early and late complications (*p* > 0.05; Table 4).

### 3.2. Quality of Life Survey

Of the 113 patients who underwent alveolar ridge reconstruction between January 2011 and December 2018, 68 were still alive during the study period. Among them, 24 agreed to complete the QoL questionnaires. Eight of the 24 patients were female (33.3%), and the mean age of these patients at the time of the survey was 59.7 years (range, 50–88 years).

Regarding the type of dental rehabilitation, six patients (25.0%) had a conventional removable prosthesis (partial and full), six (25.0%) had an implant-supported removable prosthesis, two (8.3%) had an implant-supported fixed prosthesis, and 10 (41.7%) were not wearing any prosthesis at the time of the survey. 

Regarding TNM classification, 14 patients had stage T1/T2 tumours, and 10 had T3/T4 tumours, with five patients having positive lymph node status. Four patients underwent surgical treatment alone, while the remaining 20 patients received adjuvant therapy in the form of radiotherapy (n = 8), radiochemotherapy (n = 8), radioimmunotherapy (n = 3), or a combination of these therapies (n = 1).

#### 3.2.1. OHIP-49

The median overall OHIP-49 score was 25.5 (IQR, 11.5–62.5; range, 1–115). Further study population’s OHIP-49 data can be seen in Table S1. In comparisons based on the type of dental rehabilitation, the median overall score for patients with a conventional removable prosthesis (partial or full) and those with an implant-supported prosthesis (fixed or removable) was 54 (interquartile range [IQR], 26.25–70.75) and 7 (IQR, 2–18), respectively, while the corresponding value for patients in whom no prosthetic restoration was possible was 30.5 (IQR, 13.75–75.25). 

There was no significant difference in the overall OHIP-49 score based on tumour stage (T1–T2 or T3–T4; *p* = 1.00; r = 0), lymph node invasion (N0 or N1–3; *p* = 0.89; r = 0.29), or adjuvant therapy (*p* = 0.75; η^2^ = −0.077). The problematic OHIP-49 items are shown in Table 5.

#### 3.2.2. Short Form-36

The SF-36 subscale median scores in the study population, as well as the norms for a standardised German population with tumours, are shown in Table 6 [14]. The patients of the present study showed the highest scores (100 score points) in the subscales “role limitation due to emotional problems” and “social functioning”, and “bodily pain”. The subscale “vitality” showed the lowest score (67.5 points). The medians of all subscale scores were equal to or higher than the median malignancy norm scores.

Refer to Table S2 for more study population’s SF-36 data.

**Figure 1:**
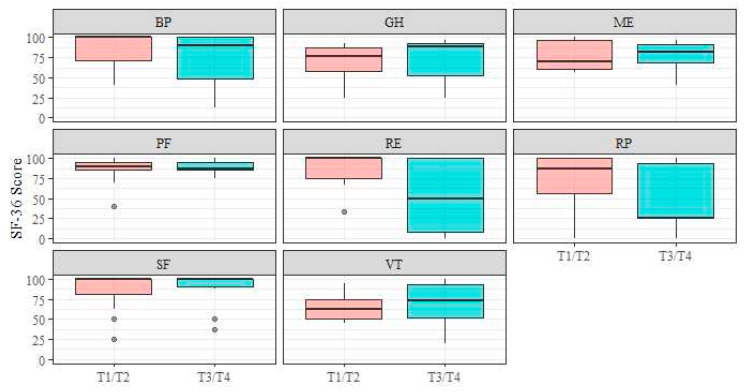
Short Form-36 subscales–tumour stage. Abbreviations: BP, bodily pain; GH, general health; ME, mental health; PF, physical functioning; RE, role limitation due to emotional problems; RP, role limitation due to physical health; SF, social functioning; VT, vitality.

No significant differences in all eight subscale scores were observed between patients with T1-T2 stage and those with T3-T4 stage (Figure 1; p_RP_ = 0.12, r_RP_ = 0.352; p_PF_ = 0.95, r_PF_ = 0.012; p_GH_ = 0.41, r_GH_ = 0.180; p_RE_ = 0.06, r_RE_ = 0.409; p_ME_ = 0.91, r_ME_ = 0.024; p_BP_ = 0.31, r_BP_ = 0.220; p_SF_ = 0.97, r_SF_ = 0.007; p_VT_ = 0.62, r_VT_ = 0.108). 

**Figure 2:**
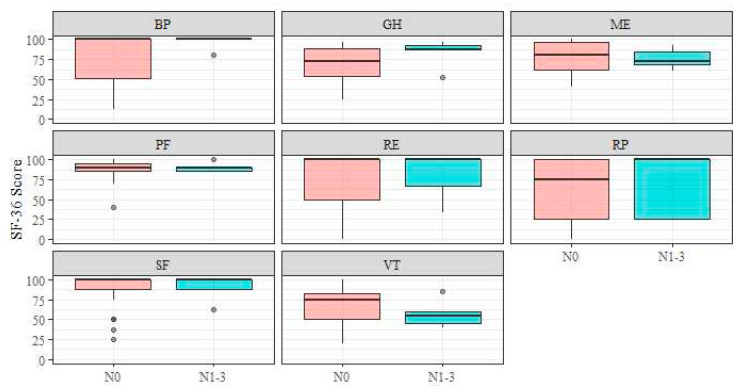
Short Form-36 subscales–N-stage. Abbreviations: BP, bodily pain; GH, general health; ME, mental health; PF, physical functioning; RE, role limitation due to emotional problems; RP, role limitation due to physical health; SF, social functioning; VT, vitality.

In addition, no significant differences were observed in relation to lymph node invasion (Figure 2; p_RP_ = 0.59, r_RP_ = 0.121; p_PF_ = 0.97, r_PF_ = 0.007; p_GH_ = 0.22, r_GH_ = 0.270; p_RE_ = 0.79, r_RE_ = 0.057; p_ME_ = 0.64, r_ME_ = 0.088; P_BP_ = 0.17, r_BP_ = 0.242; P_SF_ = 0.84, r_SF_ = 0.045; p_VT_ = 0.24, r_VT_ = 0.271). 

Adjuvant therapies, such as radiotherapy, radiochemotherapy, and radioimmunotherapy, showed no significant changes in the subscale scores in comparison with surgical therapy alone (p_RP_ = 0.84, η^2^_RP_ = −0.032; p_PF_ = 0.50, η^2^_PF_ = −0.009; p_GH_ = 0.32, η^2^_GH_ = 0.022; p_RE_ = 0.16, η^2^_RE_ = 0.117; p_ME_ = 0.50, η^2^_ME_ = 0.043; p_BP_ = 0.32, η^2^_BP_ = 0.051; p_SF_ = 0.37, η^2^_SF_ = 0.017; p_VT_ = 0.92, η^2^_VT_ = −0.039).

## 4. Discussion

### 4.1. Outcome of Dental Rehabilitation after Alveolar Ridge Reconstruction 

In this study, patients with HNC who underwent bone-free flap reconstruction of the jaw were followed up for over 10 years. Particular attention was paid to the course and success of dental rehabilitation because functional outcomes such as mastication, speech, swallowing, and psychological well-being can be severely affected by poor denture retention or the inability to wear a dental prosthesis [5,6,18]. Functional dental rehabilitation was achieved in 51.3% of the patients who underwent reconstruction. Similar results were reported by Smolka et al. in a series of 56 patients with oral cancer; in their study, 42.9% of the patients with reconstructed mandibles achieved functional dental rehabilitation [19]. However, in two other studies, only 32.2% and 31.4% of the patients with HNC achieved functional dental rehabilitation after maxillofacial reconstruction [20,21]. 

Implant-based dental rehabilitation was possible in 27.4% of our study population, which is consistent with the literature. The percentage of HNC patients with implant-retained dentures, both fixed and removable, after reconstructive surgery ranged from 24.7 to 34.8% [6,20,22]. The fact that the rate of implant-supported dental rehabilitation in HNC patients is approximately 30% is very encouraging, since functional problems may persist despite successful jaw reconstruction if prosthetic restoration is inadequate. Occasionally, an implant-supported prosthesis is the only option for masticatory restoration [5,8,10,23,24], when the vestibular space and retention capacity are inadequate for conventional prostheses after HNC treatment [9,25]. The long-term implant survival rate of 82.2% in patients was similar to that reported in other studies. The 10-year survival rates for implants after microvascular jaw reconstruction in previously reported studies were 82.0%, 79.3%, and 83.1%, respectively [6,10,26]. In some studies, the survival rate was >90% [19,20]. However, due to the differences in follow-up times, comparisons should be performed with caution since the failure rate of implants increases over time [10,25].

Ten patients lost at least one implant. It was noticeable that nine of these patients received an iliac crest transplant. The implant loss could be explained by the transplanted bone: the iliac crest has a higher proportion of cancellous bone, which may result in lower primary stability of the implants. It was also striking that 90.0% of patients with implant loss also received adjuvant radiotherapy. The corresponding percentage for patients without implant loss was 58.3%. This shows that adjuvant therapy still increases the risk of implant failure, despite precautionary measures. However, with only 10 patients, the cohort of implant losses is small, so this may also be due to random individual circumstances.

Three of the 34 patients with implant insertions remain without implant-supported prostheses. One of these three patients refused a new implant following implant loss. In the other two, dental rehabilitation was not pursued further due to a second carcinoma or recurrence.

Six of the 31 patients with implant-supported prosthetics could only eat pureed food despite successful restoration. Of note, all six patients had undergone floor of mouth and partial tongue resection. Therefore, in the absence of a functioning tongue, the inability to eat solid food was likely due to the difficulty with transporting and keeping the bolus between the dental arches. 

Both conventional and implant-supported prostheses were placed on the reconstructed jaw areas with the iliac crest flap, the fibula flap, and the scapula flap. Implant-supported prosthetics were primarily used for the medial femoral condylar flap, or dental rehabilitation was generally dispensed with. This is due to the fact that the medial femoral condylar flap is only suitable for small defects up to three centimetres due to its limited size.

The most commonly planned surgical interventions after alveolar ridge reconstruction were soft tissue corrections. These were mainly pre-prosthetic procedures, such as vestibuloplasties or aesthetic corrections, e.g., to the lips. The overall postoperative complication rate was 41.6%, which is consistent with the results of a previous study [27]. The most frequent complication (15%) requiring surgical treatment was “complications with osteosynthesis material”. The study population showed a low tumour recurrence rate of 14.1% over a long observation period of up to 10 years. The total bone flap success rate was 92.7%, similar to the findings of other reports (91.7–98.7%) [19,27,28].

Neither a higher tumour stage nor lymph node involvement was associated with an increased incidence of complications or the need for reoperation. 

### 4.2. Quality of Life Survey

The data analysis in this study was supplemented by a survey on the QoL of these patients to obtain a more comprehensive view. The treatment path for HNC with resection, microsurgical reconstruction, dental rehabilitation, and possible adjuvant therapies is long and can be stressful for the patient. Difficulties in treatment and subsequent prosthetic restoration can have functional and emotional impacts on the patient’s QoL [3,18,27].

The OHrQoL in the study population differed between the different outcomes of dental rehabilitation (median OHIP-49 score range, 5–56). In a national probability sample of 2026 German participants, the median OHIP-49 scores were between 5 and 24 [29]. Thus, oral HNC patients showed slightly worse OHrQoL after jaw reconstruction and dental rehabilitation than the standardised German norm population. However, direct comparisons should be made with caution since the study HNC population was sicker than the standardised German norm population, and the study by John et al. also included fully dentate patients.

Surprisingly, the OHrQoL score of patients without dental rehabilitation was better than that of patients with conventional tissue-borne removable prostheses. However, these results confirm the findings of previous studies: oral rehabilitation with conventional dentures is often unsatisfactory owing to altered anatomy and mucosal conditions after HNC treatment [9,25].

The OHIP-49 subscales “physical disability” and “functional limitation” were ranked as the most critical factors influencing OHrQoL in the study group, which is consistent with the results of other OHIP-49 surveys in HNC patients [30,31,32,33]. As in the present analysis, Gotfreden et al. and Fromm et al. identified the most frequently reported OHIP-49 items in their QoL surveys of patients with HNC. The following six items were found to be identical in all three studies: “difficulty chewing (Q1)”, “food catching (Q7)”, “avoid eating certain types of food (Q28)”, “trouble pronouncing words (Q2)”, “speech unclear (Q24)”, and “misunderstood some of your words (Q25)”. [30,31]. These findings emphasise that OHrQoL limitations in HNC patients are mainly caused by problems with eating and speaking. This finding was also confirmed by Lofstrand et al. in their QoL survey using the EORTC QLQ H&N35 questionnaire, in which more than 30% of the patients in the HNC subgroup had poor functioning/symptomatic related to swallowing and social eating [27]. The literature also indicates that these functional limitations can negatively affect social life. In two studies, more than 30% of the patients avoided going out in public because of eating problems [33] and reported limitations in social contact [27]. Nevertheless, we were unable to confirm these findings in our study population. However, Kumar et al. showed that normal masticatory function can be successfully achieved in patients with surgically reconstructed mandibles by rehabilitating the reconstructed site with implant-supported removable partial dentures [24]. This could explain the very good OHrQoL and patient satisfaction in our subgroup with implant-supported prostheses. 

Within the study population, six outliers consistently scored poorly on the OHIP-49 items, especially on items related to speech and mastication. Five patients underwent floor of the mouth resection with partial tongue resection and showed severe functional limitations because of the reduced and immobile tongue caused by scarring. Four patients had a percutaneous endoscopic gastrostomy tube or a percutaneous endoscopic jejunostomy tube. 

The SF-36 supplemented the oral health-specific OHIP-49 questionnaire to provide an overall, non-disease-specific view of the QoL. The results are encouraging since the HNC population had equal or even better QoL scores than the standardised German SF-36 scores for malignancies (Table 6) [14]. This, combined with the OHIP-49 results, suggests that perceived functional limitations and physical disabilities related to oral health do not significantly affect overall QoL. Earlier reports arrived at a similar conclusion [27,34].

Unexpectedly, a higher tumour stage (T3 or T4) or lymph node invasion in the study population did not influence both QoL scores. This surprising finding indicates that successful jaw reconstruction can minimise the morbidity of major resection and neck dissection, significantly improving the QoL. Similar findings were reported by Hammerlid et al., who observed no significant difference between tumour stages T1/T2 and T3/T4 in the SF-36 questionnaire [34]. In contrast, another study reported that impaired chewing and swallowing correlated significantly with an increase in tumour size and stage [35]. It should be noted that in the comparison between T1/T2 and T3/T4 cancers in the subscale “role limitation due to emotional problems”, despite the absence of statistical signifiers, both in *p*-value and effect size, a trend suggests that larger cancers are more emotionally distressing. This trend was not observed for other SF-36 subscales or the OHIP-49 questionnaire.

Additional therapies such as radiation therapy, chemotherapy, and immunotherapy did not negatively influence QoL scores in comparison with the cases without adjuvant therapy. This finding contradicts the results of previous studies that showed worse QoL in irradiated patients than in HNC patients treated only surgically [35,36].

One limitation of the QoL survey was the small sample size, with only 24 participants, and the absence of information about bias that could arise from non-response. Nevertheless, the data are representative enough to illustrate the effects of the prosthetic outcome on patients’ QoL. The authors plan a prospective multi-centre analysis.

Another limitation is the very heterogeneous group in terms of age. Since all HNC patients were included, the age range was very broad, ranging from 25.1 to 97.7 years. For this reason, age could be considered a bias for QoL, as morbidity increases with age and the ability to cope with the disease decreases. However, one study has already rejected this hypothesis, as there was no correlation between increasing age and poorer quality of life in HNC patients [37].

Overall, this study makes a significant contribution to the literature by providing a unique overview of the long-term outcome of dental rehabilitation after jaw reconstruction in HNC patients at a single centre. In addition, for the first time, insights into the impact of dental rehabilitation on the lives of individual patients were provided by combining the objective prosthetic outcome with the subjective perception through the QoL survey.

The study results and the literature analysis emphasise the importance of good communication with HNC patients concerning treatment goals. It is essential to inform patients preoperatively about the chances as well as the limitations of oral rehabilitation and how it can affect their QoL, so that they have realistic expectations.

## 5. Conclusions

Despite maximum effort to reconstruct the alveolar ridge with microvascular flaps, half of the patients remain without dental rehabilitation of the reconstructed ridge. 

Among the dental rehabilitated patients, oral health-related QoL was best in patients with implant-supported prostheses.

## Figures and Tables

**Table 1:** Treatment-specific information.

Variable	Specification	No. of Patients	Percentage
Reconstructed jaw	Maxilla	27	23.9
	Mandible	86	76.1
Microvascular flap	Iliac crest	58	51.3
	Fibula	40	35.4
	Scapula	9	8
	Femur	4	3.5
	Combination: iliac crest + femur	1	0.9
	Radius	1	0.9
Type of reconstruction	Single bone flap	41	36.3
	Composite or double flap	72	63.7
T stage	T1	13	11.5
	T2	37	32.7
	T3	19	16.8
	T4	37	32.7
	Metastasis of another carcinoma	2	1.8
	n.a.	5	4.4
N stage	N0	73	64.6
	N1	12	10.6
	N2	20	17.7
	Metastasis of another carcinoma	2	1.8
	n.a.	6	5.3

**Table 2:** Type of prostheses placed on microvascular flaps (n).

Type of Flap	Tissue Borne Denture		Implant-Supported Denture	
	Complete Denture	Partial Denture	Removable Denture	Fixed Denture
Iliac crest	11	4	8	1
Fibula	4	2	12	5
Scapula	1	3	4	1
Femur	-	-	1	1
Iliac crest + femur	-	-	-	-
Radialis	-	1	-	-

**Table 3:** Perioperative tooth extractions.

Type of Extraction	Specification	No. of Patients
Full mouth extraction		16
Partial mouth extraction	Partially dentate jaws	54
	One edentulous jaw	13
No extraction	Preoperatively edentulous	22
	Cancer in an edentulous area	8

**Table 4:** Number (percentage) of patients with reoperations/complications per subgroup.

	Complication with Osteosynthesis Material	Soft Tissue Correction	Complication with Bone Flap	Bone Flap Loss	Tumour Recurrence (Site of Origin)	Other	Tumour Recurrence (Outside Reconstruction Area)	Other (Next to Reconstruction Area)	New Bone Flap
T1	1 (7.7)	4 (30.8)	0 (0.0)	0 (0.0)	2 (15.4)	1 (7.7)	1 (7.7)	0 (0.0)	0 (0.0)
T2	9 (24.3)	12 (32.4)	6 (16.2)	4 (10.8)	2 (5.4)	2 (5.4)	1 (2.7)	2 (5.4)	4 (10.8)
T3	3 (15.8)	7 (36.8)	1 (5.3)	1 (5.3)	3 (15.8)	2 (10.5)	0 (0.0)	0 (0.0)	1 (5.3)
T4	4 (10.8)	11 (29.7)	2 (5.4)	2 (5.4)	2 (5.4)	2 (5.4)	3 (8.1)	0 (0.0)	3 (8.1)
*p*-value	0.43	0.97	0.30	0.73	0.35	0.85	0.44	0.50	0.76
N0	12 (16.4)	23 (31.5)	6 (8.2)	5 (6.8)	8 (11.0)	5 (6.8)	4 (5.5)	2 (2.7)	5 (6.8)
N1	1 (8.3)	4 (33.3)	1 (8.3)	1 (8.3)	0 (0.0)	0 (0.0)	0 (0.0)	0 (0.0)	1 (8.3)
N2	3 (15.0)	6 (30.0)	1 (5.0)	0 (0.0)	1 (5.0)	2 (10.0)	1 (5.0)	0 (0.0)	1 (5.0)
*p*-value	0.91	1.00	0.20	0.20	0.52	0.41	0.64	0.69	1.00

**Table 5:** Most frequently reported problematic items (scores 3 and 4) in OHIP-49.

OHIP-49 Item	N	Percentage	Domain
Q1 (difficulty chewing)	10	41.7%	Functional limitation
Q24 (speech unclear)	9	37.5%	Physical disability
Q2 (trouble pronouncing words)	8	33.3%	Functional limitation
Q25 (misunderstood some of your words)	8	33.3%	Physical disability
Q28 (avoid eating certain types of food)	8	33.3%	Physical disability
Q4 (appearance affected)	7	29.2%	Functional limitation
Q7 (food catching)	7	29.2%	Functional limitation
Q16 (uncomfortable eating certain type of food)	6	25.0%	Pain

**Table 6:** Short Form 36 subscale median (interquartile range) scores for the study population and German norm values.

SF-36 Subscale	Study Population	Standardised German Tumour Population ^1^
Physical functioning	90 (85–95)	80 (55–95)
Role limitation (physical health)	75 (25–100)	75 (0–100)
Role limitation (emotional problems)	100 (58.35–100)	100 (66.7–100)
Vitality	67.5 (50–81.25)	50 (35–70)
Mental health	76 (63–96)	68 (48–80)
Social functioning	100 (84.4–100)	87.5 (62.5–100)
Bodily pain	100 (62–100)	51 (31–84)
General health	83.5 (55.75–90.5)	52 (30–62)

^1^ all except skin cancer.

## Data Availability

The data presented in this study are available on request from the corresponding author.

## Supplementary Materials

The following supporting information can be downloaded at: https://www.mdpi.com/article/10.3390/jcm13113110/s1, Table S1: OHIP 49 scores’ data; Table S2: Short Form 36 subscale scores’ data.
