# Supplementary material for: Long-Term Outcomes of Dental Rehabilitation and Quality of Life after Microvascular Alveolar Ridge Reconstruction in Patients with Head and Neck Cancer
Source: J Clin Med. 2024 May 25;13(11):3110. doi: 10.3390/jcm13113110 (PMC11173157; doi:10.3390/jcm13113110)
Supplement: Supplementary file 1 [file jcm-13-03110-s001.zip › jcm-2967921-supplementary.pdf]

Table S1. OHIP 49 scores' data (n=24).

**OHIP 49**		**min**	**q1**	**median**	**mean**	**q3**	**max**	**SD**
**Total Score**		1	11.5	25.5	36	62.5	115	31.1015
**Subscale Scores**	**Functional limitation**	0	2	7.5	8.541667	13	28	7.39553
	**Handicap**	0	0	2	3.916667	7.25	16	4.96874
	**Pain**	0	0.75	2.5	4.541667	7.25	21	5.47706
	**Physical disability**	0	2.75	6	7.916667	12.25	21	6.29642
	**Psychological disability**	0	0	2	3.5	5	12	4.01085
	**Psychological discomfort**	0	0	2.5	3.833333	7	16	4.17723
	**Social disability**	0	0	0.5	2.5	5	10	3.27042

Abbreviations: min, minimum score; q1, 1st quartile; q3, 3rd quartile; max, maximum score; SD, standard deviation

Table S2. Short Form 36 subscale scores' data (n=24).

**SF-36 Subscales**	**min**	**q1**	**median**	**mean**	**q3**	**max**	**SD**
**General health**	25	55.75	83.5	72.2083333	90.5	97	22.3061293
**Role limitation due to emotional problems**	0	58.35	100	73.6125	100	100	36.7547845
**Physical functioning**	40	85	90	87.2916667	95	100	13.3497408
**Role limitations due to physical health**	0	25	75	63.5416667	100	100	38.2917623
**Mental health**	40	63	76	77.5	96	100	17.9201126
**Bodily pain**	12	62	100	80.5416667	100	100	28.9346778
**Social functioning**	25	84.375	100	86.9791667	100	100	23.4518374
**Vitality**	20	50	67.5	66.6666667	81.25	100	20.465595

Abbreviations: min, minimum score; q1, 1st quartile; q3, 3rd quartile; max, maximum score; SD, standard deviation
